# Bringing Health Policy Issues Front and Center in the Community: Expanding the Role of Community Health Coalitions^1^

**Published:** 2004-12-15

**Authors:** Joel S Meister, Jill Guernsey de Zapien

**Affiliations:** Mel and Enid Zuckerman Arizona College of Public Health; Mel and Enid Zuckerman Arizona College of Public Health, Tucson, Ariz

## Abstract

**Background:**

Systemic, environmental, and socioeconomic conditions create the context in which community members deal with their health concerns. Comprehensive, community-based chronic disease prevention interventions should address community-wide or regional policy issues that influence lifestyle behaviors associated with chronic diseases.

**Context:**

In two communities along the Arizona-Mexico border, community coalitions that administered a comprehensive diabetes prevention and control intervention expanded their membership to become policy and advocacy coalitions with broad community representation. These coalitions, or Special Action Groups (SAGs), identified and prioritized policy issues that directly or indirectly affect physical activity or nutrition.

**Methods:**

Local schools were one focus of advocacy. The Centers for Disease Control and Prevention's School Health Index was implemented as part of the overall intervention; the SAGs supported schools in advocating for more physical education programs, removal of vending machines, substitution of more healthful options in vending machines, and changes in health education curricula. In the broader community, the SAGs promoted opportunities for walking and bicycling, long-term planning by their cities and counties, and healthy food choices in local grocery stores.

Advocacy tactics included attending and making presentations at city council, school board, parks and recreation, and planning and zoning commission meetings; participating on long-range planning committees; organizing an annual community forum for elected and appointed officials; and presenting healthy food and cooking demonstrations in local markets.

**Consequences:**

After three years, SAGs were able to document changes in local policies and practices attributable to their activities.

**Interpretation:**

The SAGs contributed to systems changes in their communities and were able to obtain new resources that support protective behaviors. Also, the advocacy process itself provided strong positive reinforcement to all participants in this comprehensive diabetes intervention.

## Background

Approaches to preventing and controlling chronic diseases, such as diabetes, must focus on broad lifestyle issues. Such an approach to preventing and controlling diabetes may include patients, their families, providers, and the entire community (1-3).

More recently, and with increasing recognition of the extent to which individual health-related behavior is shaped by social and cultural norms and by the physical and policy environment of a community (4), attention is being given to the systems and environmental- and community-level factors that contribute to the behaviors that affect health status and outcomes (4-7). The Centers for Disease Control and Prevention's (CDC's) Racial and Ethnic Approaches to Community Health (REACH) 2010 program illustrates the increasing emphasis on changing systems factors using a logic model that includes changes in change agents and environmental and policy shifts as precursors of more distal changes in health-related behaviors and health status (Figure) (8).

**Figure 1:**
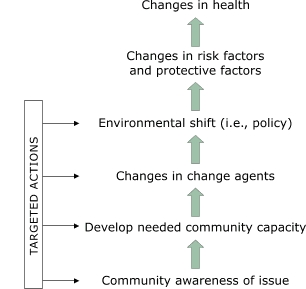
The Racial and Ethnic Approaches to Community Health (REACH) 2010 model of change, adapted by the Southwest Center for Community Health Promotion (8)

The Border Health Strategic Initiative (*Border Health ¡SI!*) was a comprehensive diabetes prevention and control program that focused on border communities along the Arizona-Mexico border (9). The authors adapted the REACH 2010 model so that *Border Health ¡SI!* included a significant policy component. The community coalitions, originally formed to bring together community partners and the University of Arizona, were challenged to become Special Action Groups (SAGs) with their own unique role — to effect policy changes that would promote health in the community.

## Context

The U.S.-Mexico border has several singular features relevant to diabetes prevention and control. It is a poor region with fragmented services, and residents often cross the border — in both directions — for health care (10). The border region has a large Hispanic population, with diabetes prevalence approximately twice the average for non-Hispanic whites (11,12). Many residents are undocumented and therefore have no access to health care except for private fee-for-service, which they can rarely afford, or for emergency services (13).

Along the Arizona-Sonora border, the University of Arizona and numerous community partners have been working together for the last twenty years to create health promotion programs and the joint capacity and infrastructure to address a wide spectrum of health issues. Based on this ongoing, evolving, and positive history of collaboration among community-based agencies and the University of Arizona and our common recognition of the need for systemic change, the partners in both communities responded positively to the recommendation that the programmatic partners of *Border Health ¡SI!* (those responsible for specific intervention components) continue to meet as a technical team while the coalition expand to include other community members and agencies with a stake in policy. These coalitions — the SAGs — would be dedicated to planning and advocating for policy change.

One of the university partners, the Cooperative Extension Service, was asked and agreed to be the facilitator for the SAGs. The Mel and Enid Zuckerman Arizona College of Public Health's collaboration with Cooperative Extension pre-dates this project, and the relationship was expanded and strengthened by the decision to have Cooperative Extension serve as SAG facilitator. As SAG facilitator, Cooperative Extension used its everyday, longstanding connection between community and university to strengthen the SAGs.

SAG membership included organizational leaders, program directors, community health workers (*promotores de salud),* and other concerned citizens. *Promotores de salud* were critical to forming SAGs (14-16). They provided the outreach and leadership in every component of the intervention except the provider component. They brought to the SAGs their knowledge of what was actually happening in the community day to day. They also provided the potential leadership for any community mobilization that might become part of the SAGs' action plans.

## Methods

### Fitting policy into the picture

SAG members met first to become familiar with the REACH 2010 model of change. The model's most novel features were emphasis on the changes in "change" agents and changes in local policies that were posited to contribute to changes in behaviors such as physical activity and nutrition. The "targeted activities" that drive the model would thus have to consist not only of the health education programs with which all partners were familiar and comfortable but with new capacity-building activities and advocacy interventions that at first seemed somewhat threatening or exotic. SAG minutes and participant observation data show that most SAG members, including organizational leaders, had never appeared before a city council or other elected body (17).

### Distinguishing between program and policy

Our community partners were highly skilled at delivering health promotion and education, but they had much less experience dealing with broader policy issues that were not part of traditional health promotion culture. These issues included, for example, the physical environment of the community and whether it supported walking or bicycle-riding or other forms of exercise, the availability of low-fat, low-sugar foods in grocery stores, the food products available in school vending machines, and the use of candy for school fundraising.

### Identifying and prioritizing policy issues

As each SAG began to identify and prioritize policy issues in its community, sustaining the distinction between programs and policies was the most challenging aspect of developing a policy agenda. For instance, in initial discussions about changing food choices, some SAG members suggested a health fair. Others, more cognizant of the policy issues, wanted to go straight to market owners or managers and attempt to influence their decisions on which food products to stock and promote and how food products were displayed.

As the policy focus became clearer, the SAGs prioritized and selected issues to be addressed over the following one to three years. Community A divided its policy goals into short- and long-term goals. The short-term goal was defined as increasing opportunities and places for physical activity, and the long-term goals were defined as making an impact on the county's long-range parks and recreation planning and resource allocation. Community B selected the following policy goals: 1) develop more parks and recreation areas, 2) work with grocery stores to offer and promote more healthful foods, and 3) work with schools to emphasize health curricula and to change the use of candy and other junk food in the fundraising and reward structure.

### Redefining health as a community-wide issue

Health came to be seen among SAG members as an array of policy issues that extend well beyond the purview of the experts in the county health department, the community health center, or school nurses. SAG members realized that they needed to reach a number of change agents that included elected officials, business people, members of the faith community, and educational leaders. They also needed to bring this broader vision to other health professionals.

### Bringing new members to the coalition

Identifying and then recruiting new SAG members was a critical step in promoting a policy agenda. Convincing some of them that health should be one of their issues was a major achievement in recruiting and retaining them as SAG members (18). These new recruits included the following (some in Community A, others in Community B): a chamber of commerce executive director, county interfaith council director, city manager, parks and recreation department director, public works department director, planning and zoning director, hospital administrator, school superintendent, town librarian, newspaper editor, and police officer.

### Developing an action plan

Once issues were identified and prioritized, the SAGs formed subgroups to develop action plans for each major issue. Community A decided to make the SAG indispensable to the county's long-range development planning effort by volunteering to serve on the planning committee, offering the SAG's own recommendations for open space, parks and recreation, and walking/bicycle paths development, and offering data gathered by its university partner.

Community A also adopted a short-term action plan that designated a three-month period for mounting a series of health promotion activities that would culminate in a presentation to the city council, stressing the need for reallocating (not increasing, at this time) parks and recreation resources to promote physical activity among the entire community, and attending to neighborhood safety, including lighting, sidewalks, and animal control. The SAG in Community A contracted with a consultant to design a compelling fact sheet that would be used in its presentation to the city council and other policy-making bodies.

In Community B, the SAG initiated an annual community forum designed to educate policy makers, advocate for policy change, and hold elected officials accountable for their support, or lack thereof, of policies to promote health. The forum was designed so that representatives of the SAG and other community groups could first present their activities and policy agendas to public officials who were invited to attend. After the community presentations, elected and appointed officials were invited to respond, and then the forum was opened to discussion.

The *promotores* in this community's SAG mobilized their constituents to advocate for new parks in one of the small towns near the border and in an unincorporated area of the county that provided few public services to its residents. These *promotores* had been leading the community walking groups and nutrition classes that were one component of *Border Health ¡SI!*. Now they and members of these groups went before the county board of supervisors to advocate for parks in their neighborhoods.

The SAG in Community B also worked with the schools component of *Border Health ¡SI!* to promote changes in the curriculum and the use of junk foods. While the schools component of *Border Health ¡SI!* worked with the School Health Index and the school health teams, the SAG also kept in close contact with the school superintendent and individual principals to promote change and monitor progress.

## Consequences

Results that can be traced directly to the actions of the SAGs are described below.

### Community A

- New walking paths were incorporated into the county's development plan.
- A new Wal-Mart Supercenter added a perimeter walking path to its construction plan.
- Plans to terminate physical education at a local school were halted.
- Health-related articles now appear regularly in the local newspaper.

### Community B

- Two Community Development Block Grants were obtained for parks and walking paths. The SAG also succeeded in convincing the local school district to donate land for one of the parks. This donation made it possible to use the grant to fund landscaping and to purchase exercise equipment and other amenities.
- Grocery stores in the target communities initiated healthy food demonstrations one to two times per month. These demonstrations were organized and conducted by *promotores*.
- Stores began stocking more healthy products.
- Sales of food featured in the healthy food demonstrations increased.
- The SAG received the 2002 Mayor's Physical Activity Leadership Award.

Of the many lessons learned from the SAGs, the following are among the most salient:

- A comprehensive approach to community health promotion requires a policy component.
- Commitment and organizational involvement of the key community-based health organizations are necessary.
- *Promotores* must be involved as change agents.
- Social action focused on policy change can energize a coalition, giving it a *raison d’être* beyond merely coordinating activities, and can contribute to its sustainability.
- The SAG created an engine for change on community health issues.
- Short-term successes contribute to long-term effectiveness of SAGs.
- Consciousness-raising about public health issues among those who are not public health practitioners is important to effecting policy change. Convincing people that health is their business regardless of what they do professionally is critical to recruiting opinion leaders to join a SAG and to activating local or regional policy makers.
- Sustainability is made possible by a SAG in several ways. SAG action motivates members to continue their advocacy efforts as new issues arise and successes are achieved. SAG advocacy creates links between programs and policies that may result in local or regional agencies incorporating successful programs and new policies into their standard mode of operation. SAGs create strategic alliances with non-health specific groups that may lead to new funding opportunities that help sustain multiple components of a community health intervention. SAGs provide an opportunity for *promotores* to serve as community change agents.

The experience of the SAGs and the results of their advocacy have been reported to the community in a variety of ways. Foremost has been the publication of numerous articles in local newspapers — made possible, no doubt, by SAG membership of newspaper editors or reporters in each community. Presentations at conferences, including the U.S.-Mexico Border Health Association, Arizona Public Health Association, CDC Diabetes Translation Conference, and others provided a mechanism for dissemination of lessons learned to other border communities throughout the region. SAG activities are also reported regularly to the Community Action Board (CAB) of the Southwest Center for Community Health Promotion. The CAB is, in effect, a super-SAG for all communities involved in *Border Health ¡SI!* and other border community health interventions of the Mel and Enid Zuckerman Arizona College of Public Health.

## Interpretation

After the fact, it is difficult to imagine the *Border Health ¡SI!* program without its SAG policy-change component. This is so not only because the SAGs contributed to systems changes in their communities and were able to obtain new resources that support protective behaviors but also because the advocacy process itself provided such strong positive reinforcement to all participants in this comprehensive diabetes intervention. The results of evaluation interviews with SAG members and the administration of the Wilder Collaboration Factors Inventory (19) strongly suggest that participation in the SAG resulted in:

- Improved health behaviors within members' own organizations.
- Better understanding of community needs.
- Closer relationships with other agencies represented on the SAGs.

SAG members also took credit for:

- Building awareness among policy makers.
- Influencing community-wide resource allocation.
- Gaining support for SAG initiatives by city, county, and school-governing bodies.
- Working collaboratively with decision makers in the planning process.

Context always plays an important role in defining the issues to be addressed and the boundaries of possible action and change in a given community. In this case, context included the border geography and demography, especially the preponderance of Hispanics in these communities, the persistent poverty and lack of formal education among much of the population, and the pervasiveness of diabetes. One might suppose that such a context would militate against effective organization for policy change. We did not find this to be true. On the contrary, the brief history of the SAGs confirms our prior experience — that in these have-not communities along the U.S.-Mexico border, there is a largely untapped reservoir of intelligence and thirst for knowledge, concern about community conditions, desire for change and willingness to take risks, and, most important, a willingness to act collectively for the common good.

From the perspective of university-based participatory-action researchers, creating a collaborative policy-change initiative, whether stand-alone or as part of a broad health intervention, requires a strong, positive university-community partnership (20,21). Those partnerships take time to build and require mutual trust (22-25). To that we would add that the researcher's goal is to be a partner in the fullest sense, not merely to provide technical assistance, advise, and evaluate but to be an integral part of planning, decision making, and action — without inadvertently assuming the leadership of what is, after all, a community coalition. It is the action taken by *all* of the partners that results in the kind of impact that lives on in the community.
